# Migratory birds in southern Brazil are a source of multiple avian influenza virus subtypes

**DOI:** 10.1111/irv.12519

**Published:** 2017-12-15

**Authors:** Jansen Araujo, Maria Virgínia Petry, Thomas Fabrizio, David Walker, Tatiana Ometto, Luciano M. Thomazelli, Angelo L. Scherer, Patricia P. Serafini, Isaac S. Neto, Scott Krauss, Robert G. Webster, Richard J. Webby, Edison L. Durigon

**Affiliations:** ^1^ Laboratório de Virologia Clínica e Molecular do Instituto de Ciências Biomédicas (ICB‐II) Universidade de São Paulo São Paulo SP Brazil; ^2^ Laboratório de Ornitologia e Animais Marinhos (LOAM) Universidade do Vale do Rio dos Sinos, UNISINOS São Leopoldo RS Brazil; ^3^ Department of Infectious Diseases St. Jude Children's Research Hospital Memphis TN USA; ^4^ Centro Nacional de Pesquisa e Conservação das Aves Silvestres (CEMAVE/ICMBio/MMA), Brazil Florianópolis Brazil

**Keywords:** avian influenza virus, Brazil, clade, H6N1, migratory birds, North America, reassortment, South America, waterfowl

## Abstract

**Background:**

There is insufficient knowledge about the relation of avian influenza virus (AIV) to migratory birds in South America. Accordingly, we studied samples obtained over a 4‐year period (2009‐2012) from wild birds at a major wintering site in southern Brazil.

**Methods:**

We obtained 1212 oropharyngeal/cloacal samples from wild birds at Lagoa do Peixe National Park and screened them for influenza A virus by RT‐PCR amplification of the matrix gene. Virus isolates were subjected to genomic sequencing and antigenic characterization.

**Results:**

Forty‐eight samples of 1212 (3.96%) contained detectable influenza virus RNA. Partial viral sequences were obtained from 12 of these samples, showing the presence of H2N2 (1), H6Nx (1), H6N1 (8), H9N2 (1), and H12N5 (1) viruses. As H6 viruses predominated, we generated complete genomes from all 9 H6 viruses. Phylogenetic analyses showed that they were most similar to viruses of South American lineage. The H6N1 viruses caused no disease signs in infected ferrets and, despite genetic differences, were antigenically similar to North American isolates.

**Conclusions:**

Lagoa do Peixe National Park is a source of multiple AIV subtypes, with the levels of influenza virus in birds being highest at the end of their wintering period in this region. H6N1 viruses were the predominant subtype identified. These viruses were more similar to viruses of South American lineage than to those of North American lineage.

## 1 INTRODUCTION

Aquatic birds, particularly the Anseriformes (waterfowl) and Charadriiformes (gulls and shorebirds), are the natural reservoirs of avian influenza virus (AIV) and serve to perpetuate most known influenza A subtypes.^1^, ^2^, ^3^ The major migratory bird stopover sites may create areas for enhanced interspecies contact, AIV transmission, and AIV genetic reassortment in different bird species from different flyways.^4^ Every year, millions of wild birds from different North American flyways converge at wintering sites in Brazil. The Lagoa do Peixe National Park is recognized as the most important of these stopovers and acts as a wintering site for North American shorebirds.^5^, ^6^, ^7^ The birds usually remain in Brazil from September to May, and they depend on this habitat to replenish the energy expended in their winter migration.^8^ Lagoa do Peixe National Park is also important to resident birds and to South American migrant birds,^9^ suggesting that there is great potential there for the spread and genetic reassortment of influenza A viruses. Despite the commingling of birds from major North and South American flyways, there is a marked phylogenic separation of some influenza viruses residing within them. Based on genetic sequence, there are 2 major groups of influenza viruses in the Americas, one made up of viruses from North and South America, and the second only with viruses from South America.^10^

Influenza A viruses are composed of 8 RNA segments and are subtyped according to their hemagglutinin (HA) and neuraminidase (NA) antigenic glycoproteins. Evidence suggests that viruses representing the 16 HA and 9 NA subtypes exist in harmony with their natural reservoirs, causing little or no disease.^11^ AIVs in Eurasia and Australia appear to have diverged from those in North America, presumably as a result of different flyways being established and a consequent lack of contact between birds and therefore viruses, from the different continents.^12^ Similarly, the HA and NA genes of AIVs in Argentina differ from those of viruses circulating in North America and Eurasia.^13^ The main subtypes of AIV consistently reported to circulate in other countries^14^, ^15^, ^16^, ^17^, ^18^, ^19^ have not been reported in Brazil. However, an H11N9 influenza virus was recently identified for the first time in South American migratory birds in the Amazon region, and this virus showed great similarity to viruses of North American, rather than South American, lineages.^20^

Compared to surveillance activities in other regions of the world, influenza virus surveillance in South American wildlife has been limited. It remains unclear whether the North American and South American AIV lineages converge and whether migrating wild birds transport AIVs of South American lineage to the United States.^21^ Much remains unknown about AIV ecology in Brazil, including the identity of the main virus carrier species and the extent to which wild birds support virus spread between North and South America. Here, we studied samples obtained from wild birds in southern Brazil over a 4‐year period to determine the extent of AIV circulation, the major host species of AIV, and the nature of any viruses present.

## 2 METHODS

### 2.1 Ethics statement

The study was conducted in strict accordance with the guidelines of the Ethical Principles of Animal Experimentation adopted by the Brazilian Society of Laboratory Animal Science (SBCAL), and the animal protocol was approved by the Ethics Committee on Animal Experiments (EAEC) (Permit No. 105). All procedures involving wild birds were approved by the Ministério do Meio Ambiente (MMA) and the Instituto Chico Mendes de Conservação da Biodiversidade (ICMBio/SISBIO) (SISBIO numbers. 17565‐1, 22976‐8, 23159‐3, 24381‐3, and 14966‐1). The ferret studies were approved by the Institutional Animal Care and Use Committee of St. Jude Children's Research Hospital.

### 2.2 Study site and sample collection

**Figure 1:**
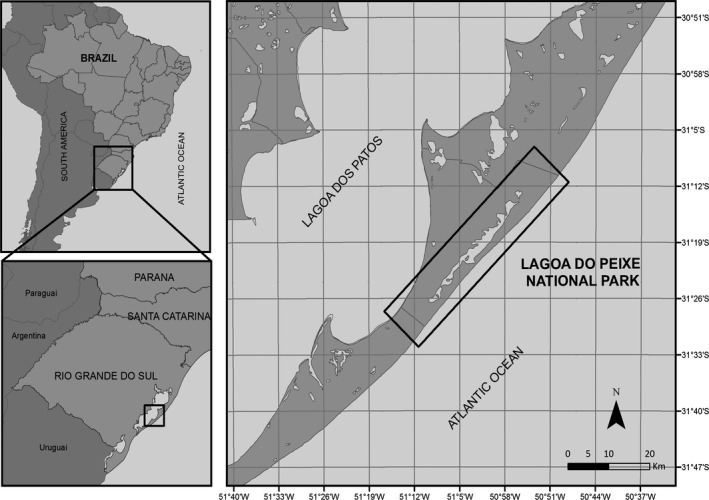
Sampling site in Lagoa do Peixe National Park. The inset indicates the location of Rio Grande do Sul State in Brazil

During the years 2009 through 2012, AIV surveillance was conducted in Lagoa do Peixe National Park in Rio Grande do Sul State, Brazil (31°82′17.94′′S, 51°80′52.37″W), which is a natural gathering site for migratory birds (Figure 1). Oropharyngeal and cloacal swabs were obtained from 1212 wild birds. The bird families captured in mist nets included Ardeidae, Charadriidae, Haematopodidae, Recurvitostridade, Scolopacidae, Rostratulidae, Tyrannidae, Furnariidae, and Laridae, except Kelp Gull, and were then released after sampling. The other bird families were sampled along a 120‐km stretch of beach north of the Lagoa do Peixe National Park, between Balneário Pinhal (lat 30°14′55″S, long, 50°13′47″W) and Mostardas (lat 31°10′52″S, long 50°50′03″W). Oropharyngeal and cloacal samples were also collected from recently stranded dead birds on the beach in monthly trips from November 2009 to June 2012. The swabs were placed in vials containing VTM transport medium composed of salt solution (PBS) supplemented with antimicrobial agents (200 U/mL penicillin G, 200 U/mL streptomycin, 25 μg/mL fungizone, and 6 μg/mL gentamycin) and 10% glycerol. All samples were immediately placed in liquid nitrogen following collection in duplicate. The duplicate sample was shipped to St. Jude Children's Research Hospital via the vapor phase of liquid nitrogen to prevent any freeze/thaw cycles.

### 2.3 RNA extraction and AIV screening

RNA was extracted from the VTM medium using an RNA extraction kit (MagMax TM‐96 RNA Isolation Kit; Ambion, Austin, TX, USA) in accordance with the manufacturer's instructions. The RNA was eluted into 80 μL of RNase‐free water. All samples were screened for the presence of the AIV matrix gene by one‐step real‐time reverse transcriptase (RT)‐PCR, using an AIV‐M TaqMan Reagents Kit (Applied Biosystems, Foster City, CA, USA).^20^, ^22^

### 2.4 Virus isolation and subtyping

Virus was isolated by standard methods in 10‐day‐old embryonated chicken eggs, and viral RNA was amplified from samples positive for AIV by real‐time RT‐PCR.^23^, ^24^ The Flu DETECT Avian Influenza Virus Type A Test Kit (Synbiotics, San Diego, CA, USA) was used to quickly confirm the presence of AIV protein within each sample.^25^ Hemagglutination and neuraminidase inhibition tests were performed with a panel of polyclonal reference sera to identify the influenza subtypes as recorded in Table 1.^26^, ^27^, ^28^

**Table 1:** Forty‐eight samples testing positive for influenza A virus. The 17 samples in which the presence of a specific subtype was confirmed are in bold. Nine H6N1‐subtype viruses were confirmed, along with one virus of each of the H6Nx, H2N2, H12N5, and H9N2 subtypes

Tubes	Sample ID	Ct	Scientific name	Date	Location	HA	Flu detect	HI	Subtype
1	PNLP 233	38	*Calidris fuscicollis*	25/03/2010	Lagoa do Peixe	−			–
**2**	**PNLP 304**	**37**	***Sterna hirundo***	**27/03/2010**	**Lagoa do Peixe**	**−**	** **	** **	**H6N**
**3**	**PNLP 315**	**35**	***Calidris canutus***	**27/03/2010**	**Lagoa do Peixe**	**+**	**+**	**640**	**H12N5**
4	PNLP 319	35	*C. canutus*	27/03/2010	Lagoa do Peixe	−			–
5	PNLP‐320	37	*C. canutus*	27/03/2010	Lagoa do Peixe	−			–
6	PNLP‐325	36	*C. fuscicollis*	27/03/2010	Lagoa do Peixe	−			–
7	PNLP‐346	37	*C. fuscicollis*	27/03/2010	Lagoa do Peixe	−			–
8	PNLP‐395	39	*C. fuscicollis*	29/03/2010	Lagoa do Peixe	−			–
9	RS 738	36	*Larus dominicanus*	20/12/2010	Lagoa do Peixe	−			–
**10**	**RS 787**	**38**	***C. canutus***	**04/04/2011**	**Lagoa do Peixe**	**−**	** **	** **	**H9N2**
**11**	**RS 1147**	**28**	***C. fuscicollis***	**17/04/2012**	**Lagoa do Peixe**	**−**	** **	** **	**H6N1**
12	RS 1148	36	*Charadrius semipalmatus*	17/04/2012	Lagoa do Peixe	−			–
**13**	**RS 1149**	**25**	***C. fuscicollis***	**17/04/2012**	**Lagoa do Peixe**	**+**	**+**	**160**	**H6N1**
14	RS 1150	34	*C. fuscicollis*	17/04/2012	Lagoa do Peixe	−			–
**15**	**RS 1151**	**30**	***C. fuscicollis***	**18/04/2012**	**Lagoa do Peixe**	**+**	**+**	**640**	**H6N1**
16	RS 1152	39	*C. semipalmatus*	18/04/2012	Lagoa do Peixe	−			–
17	RS 1153	38	*C. semipalmatus*	18/04/2012	Lagoa do Peixe	−			–
**18**	**RS 1154**	**29**	***C. fuscicollis***	**18/04/2012**	**Lagoa do Peixe**	**−**	** **	** **	**H6N1**
19	RS 1155	32	*C. fuscicollis*	18/04/2012	Lagoa do Peixe	−			–
20	RS 1156	34	*C. fuscicollis*	18/04/2012	Lagoa do Peixe	−			–
21	RS 1158	31	*C. fuscicollis*	18/04/2012	Lagoa do Peixe	−			–
**22**	**RS 1167**	**32**	***C. fuscicollis***	**18/04/2012**	**Lagoa do Peixe**	**+**	**−**	**–**	**H6N1**
**23**	**RS 1169**	**33**	***C. fuscicollis***	**18/04/2012**	**Lagoa do Peixe**	**−**	** **	** **	**H6N1**
24	RS 1170	38	*C. fuscicollis*	18/04/2012	Lagoa do Peixe	−			–
**25**	**RS 1177**	**23**	***C. fuscicollis***	**18/04/2012**	**Lagoa do Peixe**	**+**	**−**	**–**	**H6N1**
26	RS 1179	39	*C. fuscicollis*	18/04/2012	Lagoa do Peixe	−			–
27	RS 1183	38	*C. fuscicollis*	18/04/2012	Lagoa do Peixe	−			–
28	RS 1188	32	*C. fuscicollis*	18/04/2012	Lagoa do Peixe	−			–
29	RS 1189	38	*C. fuscicollis*	18/04/2012	Lagoa do Peixe	−			–
30	RS 1190	39	*C. fuscicollis*	18/04/2012	Lagoa do Peixe	−			–
**31**	**RS 1193**	**30**	***C. fuscicollis***	**18/04/2012**	**Lagoa do Peixe**	**−**	** **	** **	**H2N2**
**32**	**RS 1196**	**30**	***C. fuscicollis***	**18/04/2012**	**Lagoa do Peixe**	**−**	** **	** **	**H6N1**
33	RS 1197	39	*C. semipalmatus*	18/04/2012	Lagoa do Peixe	−			–
34	RS 1198	39	*Rynchops niger*	18/04/2012	Lagoa do Peixe	−			–
35	RS 1199	40	*C. fuscicollis*	18/04/2012	Lagoa do Peixe	−			–
36	RS 1200	38	*C. fuscicollis*	18/04/2012	Lagoa do Peixe	−			–
37	RS 1203	38	*C. fuscicollis*	19/04/2012	Lagoa do Peixe	−			–
38	RS 1204	35	*C. fuscicollis*	19/04/2012	Lagoa do Peixe	−			–
39	RS 1205	36	*C. fuscicollis*	19/04/2012	Lagoa do Peixe	−			–
40	RS 1207	39	*C. fuscicollis*	19/04/2012	Lagoa do Peixe	−			–
41	RS 1208	35	*C. fuscicollis*	00/01/1900	Lagoa do Peixe	+	−	NDV:160	–
42	RS 1210	38	*C. fuscicollis*	19/04/2012	Lagoa do Peixe	−			–
43	RS 1212	39	*Haematopus palliatus*	19/04/2012	Lagoa do Peixe				–
44	RS 1216	39	*C. fuscicollis*	19/04/2012	Lagoa do Peixe	−			–
45	RS 1217	33	*C. fuscicollis*	19/04/2012	Lagoa do Peixe	−			–
46	RS 1218	39	*Calidris alba*	19/04/2012	Lagoa do Peixe	−			–
47	RS 1222	33	*R. niger*	19/04/2012	Lagoa do Peixe	−			–
48	RS 1239	37	*C. fuscicollis*	22/04/2012	Lagoa do Peixe	−			–
**49**	**i536 DE**	**–**	**Shorebirds**	**2012**	**Delaware Bay**	**+**	**+**	**320**	**H6N1**
**50**	**i548 DE**	**–**	**Shorebirds**	**2012**	**Delaware Bay**	**+**	**+**	**640**	**H6N1**
**51**	**i556 DE**	**–**	**Shorebirds**	**2012**	**Delaware Bay**	**+**	**+**	**512**	**H6N1**
**52**	**i558 DE**	**–**	**Shorebirds**	**2012**	**Delaware Bay**	**+**	**+**	**512**	**H6N1**
**53**	**i595 DE**	**–**	**Shorebirds**	**2012**	**Delaware Bay**	**+**	**+**	**512**	**H6N1**

Ct, real‐time RT‐PCR cycle threshold; HA, hemagglutination assay; HI, hemagglutination inhibition assay.

### 2.5 Virus sequencing

Viral RNA was extracted from 200 μL of allantoic fluid using the RNeasy Mini Kit (Qiagen, Hilden, Germany) in accordance with the manufacturer's instructions. RNA was eluted into 80 μL of RNase‐free water. The cDNA was transcribed using a High‐capacity cDNA Archive Kit (Applied Biosystems) in accordance with the manufacturer's directions. The full genomes of all H6‐positive samples were sequenced using a high‐throughput next‐generation sequencing pipeline on an Ion Torrent Personal Genome Machine (PGM) sequencer (Life Technologies Inc., New York, NY, USA)^29^, ^30^, ^31^ in a BSL3^+^ laboratory at the University of São Paulo and on Roche 454 GS FLX (Roche Diagnostics Corp., Branford, CT, USA) and Illumina MiSeq platforms (Illumina, San Diego, CA, USA), as described previously,^32^, ^33^, ^34^ at St. Jude Children's Research Hospital. Samples for MiSeq sequencing were prepared using the Illumina Nextera XT DNA Library Preparation Kit in accordance with the manufacturer's instructions. Briefly, whole influenza genomes were amplified using universal primers (sequences available upon request) and tagmented into fragments of approximately 300 bp each. The fragments were enriched and amplified before sequencing on the MiSeq instrument using the 300‐cycle MiSeq Reagent v2 Kit (Illumina). Sequences were deposited in the GenBank database under accession numbers KX620053‐KX620135, KX670559‐KX670562, KX814376‐KX814382, and KX827310.

### 2.6 Phylogenetic analysis

The complete sequences of all segments of each isolate were compared using the Basic Local Alignment Search Tool (BLAST) to identify the most closely related sequences available in public databases.^35^ The nucleotide sequences of each gene segment were aligned using clc genomics workbench version 7.6 (Qiagen, Aarhus, Denmark), and the percentage nucleotide similarity was calculated using the MegAlign program of the dnastar package (DNASTAR, Inc., Madison, WI, USA). The available full‐genome reference sequences of AIVs isolated from birds were used in the construction of the phylogenetic trees. The HA and NA genes were analyzed using open reading frames (ORFs); to identify the most closely related sequences, maximum likelihood trees were inferred on the basis of nucleotide alignment, using the program Bayesian Markov Chain Monte Carlo (MCMC) in v1.8.0,^36^ with the generalized time‐reversible (GTR) model and gamma‐distributed rates among sites.

### 2.7 Ferret studies

To prepare post‐infection ferret antiserum, ferrets aged approximately 8 months (Triple F Farms, Sayre, PA, USA), previously verified as seronegative for influenza A (H1 and H3 subtypes) and influenza B by hemagglutinin inhibition (HI) tests, were inoculated with 1:10 dilutions of each viral stock in a 0.5‐mL volume under light isoflurane anesthesia (actual doses ranged from 10^4.9^ to 10^6.4^). After 21 days, blood was collected to test for seroconversion by HI assay with turkey red blood cells.^37^, ^38^ The HI assay was conducted in triplicate, and the titers are reported as the geometric means. An HI titer of 40 or higher is indicative of seroconversion. All ferret studies were conducted in an ABSL‐2 laboratory space in accordance with the St. Jude Children's Research Hospital Animal Care and Use Committee guidelines.

## 3 RESULTS

### 3.1 Virus detection

We examined 1212 samples from wild birds. Cloacal and oropharyngeal swabs from each bird were collected and combined to facilitate the analysis (Table 2). Each year of the study featured 2 distinct events: the arrival of birds from the Northern Hemisphere in November/December (at the end of their migration south) and their departure in March/April (for their migration north). Most of the samples were collected from migratory bird species. The AIV M gene was detected in 48 of 1212 samples (3.96%), most of which came from White‐rumped sandpipers (*Calidris fuscicollis*). With the exception of one sample collected in December 2010, all of the positive samples were collected during March or April, suggesting that the AIV prevalence builds up during the wintering period and that birds migrating northward carry a burden of virus with them. We detected the M gene at a higher rate in 2012 (9/163 birds, 5.5%) than in 2010 (2/371 birds, 0.53%) or 2011 (1/466 birds, 0.21%); no M gene was detected in 2009 (0/212 birds). The lack of positive samples collected during the austral summer also suggests that AIV is not maintained at high levels in resident bird populations.

**Table 2:** Frequency of AIV RNA detection by bird family and by species sampled at Lagoa do Peixe National Park, southern Brazil. Status: SA, South American migrant; NA, North American migrant; R, resident

Family	Scientific name	Common name	Status	Sampling	Positives	AIV frequency (%)	AIV subtype
Spheniscidae	*Spheniscus magellanicus*	Magellanic penguin	SA	151	0	–	–
Procellariidae	*Thalassarche chlororhynchos*	Atlantic yellow‐nosed albatross	SA	15	0	–	–
	*Thalassarche melanophrys*	Black‐browed albatross	SA	21	0	–	–
	*Macronectes giganteus*	Southern giant petrel	SA	6	0	–	–
	*Pterodroma mollis*	Soft‐plumaged petrel	SA	1	0	–	–
	*Pterodroma incerta*	Atlantic petrel	SA	2	0	–	–
	*Pachyptila desolata*	Antarctic prion	SA	1	0	–	–
	*Pachyptila belcheri*	Slender‐billed prion	SA	1	0	–	–
	*Procellaria aequinoctialis*	White‐chinned petrel	SA	18	0	–	–
	*Calonectris borealis*	Cory's shearwater	NA	21	0	–	–
	*Ardenna gravis*	Great shearwater	SA	17	0	–	–
	*Puffinus puffinus*	Manx shearwater	NA	39	0	–	–
Oceanitidae	*Oceanites oceanicus*	Wilson's storm‐petrel	SA	1	0	–	–
Phalacrocoracidae	*Phalacrocorax brasilianus*	Neotropic cormorant	R	4	0	–	–
Threskiornithidae	*Plegadis chihi*	White‐faced ibis	R	1	0	–	–
Ardeidae	*Egretta thula*	Snowy egret	R	2	0	–	–
Accipitridae	*Buteo* sp.	Hawks	–	1	0	–	–
	*Buteogallus meridionalis*	Savanna hawk	R	1	0	–	–
Rallidae	*Fulica leucoptera*	White‐winged coot	R	1	0	–	–
	*Gallinula galeata*	Common gallinule	R	1	0	–	–
Charadriidae						5.26	
	*Vanellus chilensis*	Southern lapwing	R	6	0	–	–
	*Pluvialis dominica*	American golden plover	NA	31	0	–	–
	*Pluvialis squatarola*	Black‐bellied plover	NA	6	0	–	–
	*Charadrius semipalmatus*	Semipalmated plover	NA	28	4	14.29	
	*Charadrius collaris*	Collared plover	R	3	0	–	–
	*Charadrius falklandicus*	Two‐banded plover	SA	1	0	–	–
	*Charadrius modestus*	Rufous‐chested plover	SA	1	0	–	–
Haematopodidae						3.13	
	*Haematopus palliatus*	American oystercatcher	R	32	1	3.13	
Recurvitostridade	*Himantopus melanurus*	White‐backed stilt	R	35	0	–	–
Scolopacidae	* *					7.41	
	*Tringa melanoleuca*	Greater yellowlegs	NA	5	0	–	–
	*Tringa flavipes*	Lesser yellowlegs	NA	10	0	–	–
	*Arenaria interpres*	Ruddy turnstone	NA	10	0	–	–
	*Calidris canutus*	Red knot	NA	44	4	9.09	H12N5 (1); H9N2 (1)
	*Calidris alba*	Sanderling	NA	25	1	4.00	
	*Calidris pusilla*	Semipalmated sandpiper	NA	14	0	–	–
	*Calidris fuscicollis*	White‐rumped sandpiper	NA	370	34	9.19	H6N1 (8); H2N2 (1)
	*Calidris melanotos*	Pectoral sandpiper	NA	1	0	–	–
	*Calidris himantopus*	Stilt sandpiper	NA	2	0	–	–
	*Calidris subruficollis*	Buff‐breasted sandpiper	NA	45	0	–	–
Rostratulidae	*Nycticryphes semicollaris*	South American painted snipe	R	2	0	–	–
Laridae						1.71	
	*Larus maculipennis*	Brown‐hooded gull	R	4	0	–	–
	*Larus dominicanus*	Kelp gull	R	4	1	25.00	
	*Sternula superciliaris*	Yellow‐billed tern	R	7	0	–	–
	*Sterna hirundo*	Common tern	NA	83	1	1.20	H6Nx (1)
	*Sterna hirundinacea*	South American tern	R	9	0	–	–
	*Sterna vittata*	Antarctic tern	SA	1	0	–	–
	*Sterna trudeaui*	Snowy‐crowned tern	R	13	0	–	–
	*Thalasseus acuflavidus*	Cabot's tern	R	3	0	–	–
	*Rynchops niger*	Black skimmer	R	110	2	1.82	
Tyrannidae	*Elaenia mesoleuca*	Olivaceous elaenia	R	1	0	–	–
Furnariidae	*Anumbius annumbi*	Firewood‐gatherer	R	1	0	–	–
Total				1212	48	3.96	

### 3.2 Virus identification

Of the 48 samples positive for the M gene, 12 yielded enough sequence data for subtyping the virus. The subtypes identified were H2N2 (1), H6Nx (1), H6N1 (8), H9N2 (1), and H12N5 (1). The samples containing H6NX virus from the common tern (*Sterna hirundo*) and H12N5 virus from the red knot (*Calidris canutus*) were collected in the 2010 austral autumn; the sample containing H9N2 (*C. canutus*) was collected in the 2011 austral autumn, and the samples containing H6N1 and H2N2 viruses were collected in the 2012 austral autumn. All 8 of the H6N1 viruses from 2012 were isolated from samples collected from White‐rumped sandpipers. We attempted to isolate virus from all M gene–positive samples but recovered only 2 of the H6N1 viruses collected in 2012 and only one H12N5 virus collected in 2010.

### 3.3 Genetic and antigenic analyses of isolated H6N1 viruses

**Figure 2:**
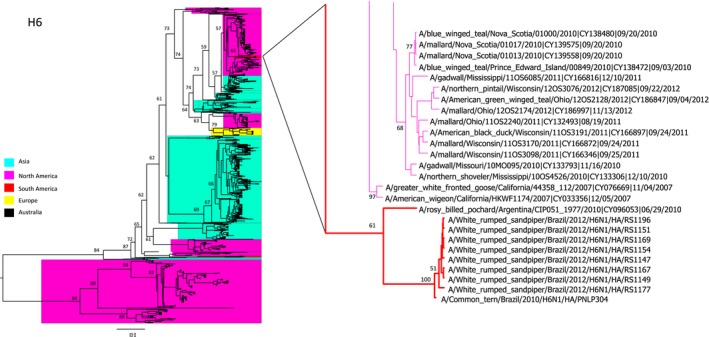
Phylogenetic analysis of influenza A virus based on the hemagglutinin gene. The trees are drawn to similar scales with branch lengths proportional to the evolutionary distance. The clusters of sequences from the Brazilian samples are indicated by branches colored red. Bootstraps values >50% were obtained in the analysis of 1000 replicates and are presented at the branching points

**Figure 3:**
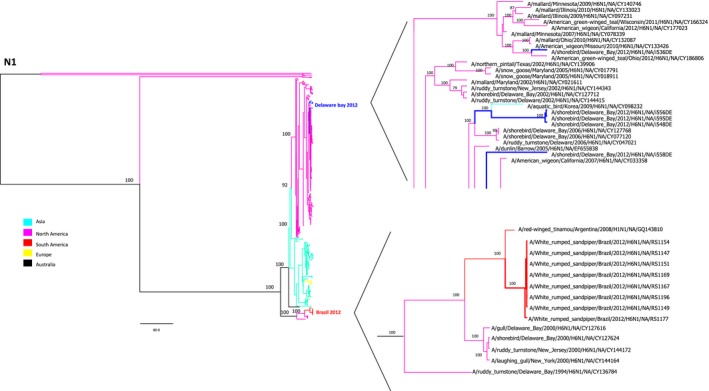
Phylogenetic analysis of influenza A virus based on the neuraminidase gene. The trees are drawn to similar scales with branch lengths proportional to the evolutionary distance. Bootstraps values >50% were obtained in the analysis of 1000 replicates and are presented at the branching points. The clusters of sequences from the Brazilian samples are indicated by branches colored red. The clusters of sequences from Delaware Bay, isolated contemporaneously in the United States, are indicated by branches colored blue

**Figure 4:**
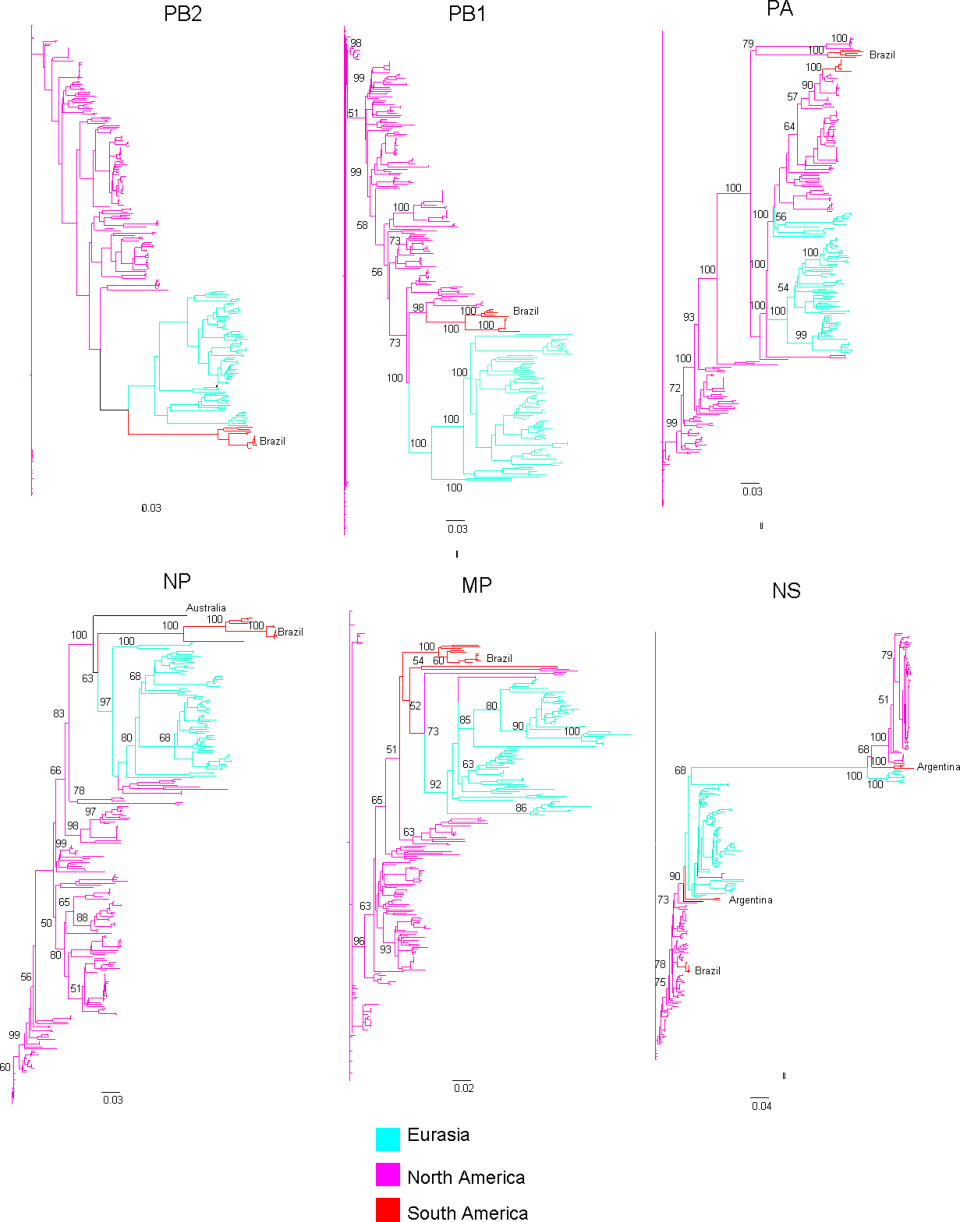
Phylogenetic analysis of influenza A virus based on internal gene sequences. The trees are drawn to similar scales with branch lengths proportional to evolutionary distance. Clades are broadly classified as Eurasian (green), North American (pink), and South American (red). Bootstraps values >50% were obtained in the analysis of 1000 replicates and are presented at the branching points. The phylogenetic trees were constructed using maximum likelihood methodology. Detailed phylogenetic trees with virus designations can be found in Figure [Link], [Link], [Link], [Link], [Link], [Link] in the Supporting Information

In our genetic analysis, we were able to generate complete genomes from 9 samples. Eight of these genomes were obtained from swab material, and only one was obtained from an egg isolate. All 9 viruses had >99% amino acid homology with each other. A comparison of the virus surface glycoprotein gene sequences by BLAST analyses showed the HA glycoproteins of all the viruses to be similar to those of the H6 subtype (with 97% to 99% similarity), and their NA glycoproteins were similar to those of the N1 subtype (with 96% to 99% similarity). Both the HA and NA genes showed a strong relation to South American H6N1 lineages, as did their other genes (Figures 2, 3, 4).

The phylogenetic analysis used 1013 sequences available in the GenBank database, together with 5 new sequences of H6 viruses isolated the same year in Delaware Bay (North America) for comparison. The data showed that the South American isolates formed a unique clade; however, it was still similar to the North American viruses (Figures 2 and 3). Although the viruses from Brazilian samples were closely related to North American sequences, they formed a divergent clade consisting of only South American H6N1 sequences.

To assess the antigenic relatedness of our isolated H6N1 viruses to North American H6N1 viruses, we infected ferrets with each virus at various doses. Although we did not power the ferret study to assess pathogenicity or transmission, none of the infected ferrets showed any overt clinical signs, and all of them remained healthy and active throughout the study. HI analysis showed that the South and North American H6N1 viruses were antigenically indistinguishable (Table 3).

**Table 3:** HI analysis of North American and South American H6N1 viruses. The HI assays were conducted in triplicate, and the titers are reported as the geometric mean. An HI titer of 40 or higher is indicative of seroconversion

	Sandpiper/Brazil/RS1149		Sandpiper/Brazil/RS1151		Shorebird/DE/548		Shorebird/DE/595	
	F‐930	F‐928	F‐925	F‐926	F‐921	F‐922	F‐919	F‐920
A/White‐rumped sandpiper/Brazil/RS1149/2012	80	80	40	31.75	40	80	127	63.5
A/Shorebird/DE/548/2012	80	80	80	40	80	80	254	80
A/Shorebird/DE/595/2012	80	80	40	31.75	40	80	160	80

## 4 DISCUSSION

In our 4 years of sampling at this important migratory bird wintering site, we found evidence of AIV circulation in consecutive years, with H6N1 viruses being predominant (mostly as a result of increased detection in the austral autumn of 2012). Rimondi et al^13^ reported the existence of a South American H6 influenza virus lineage in Argentinian aquatic birds in 2008; most of the Argentinian virus genes were phylogenetically distinct from those of the North American lineages and had probably evolved independently and in isolation. However, one of the sequenced NA genes appeared to be derived from a virus of North American lineage, indicating that viruses of the 2 American genetic lineages do have opportunities to interact.^13^

The H6 subtype isolated in our study showed high sequence similarity to the Argentinian H6 virus isolated from a Rosy‐Billed Pochard (*Netta peposaca*). This result is difficult to explain. The birds we sampled are known to migrate to North America, and we identified the highest prevalence of virus just before this northward migration, yet the viruses we isolated were clearly distinct from those detected in North America. These data suggest that the viruses isolated in the austral autumn do not reach North America but are cleared from this population of birds en route. Our data also support a hypothesis that the source of AIV infection at Lagoa do Peixe National Park is local resident birds (or birds migrating to this area from other regions of South America), rather than birds migrating to the area from North America. We should note that 2012 saw the highest recorded incidence of virus detection in wild birds at Lagoa do Peixe, perhaps reflecting the great number of White‐rumped sandpipers (*C. fuscicollis*) that congregated there that year. This species is among the most abundant of the shorebird species that migrate to South America. White‐rumped sandpipers migrate from the Arctic to the Nova Scotia coast and then follow the Atlantic route to Patagonia, stopping over in Colombia, Venezuela, Suriname, and South Brazil. During the austral summer, flocks of up to 7000 White‐rumped sandpipers use the Lagoa do Peixe National Park as a feeding, molting, and resting area. The flocks are greatest there during April/May, when individuals wintering in Patagonia fly north to join those that winter in Lagoa do Peixe National Park for feeding and remain there until migrating back to North America. In wintering and stopover areas in South America, the species may be found along with other migratory shorebirds from North America [eg, the red knot (*C. canutus)*, sanderling (*Calidris alba)*, and semipalmated plover (*Charadrius semipalmatus*)], as well as South American migrants [eg, the black skimmer (*Rynchops niger*) and Chilean flamingo (*Phoenicopterus chilensis*)] and resident species [eg, the American oystercatcher (*Haematopus palliates*), southern lapwing (*Vanellus chilensis*), and collared plover (*Charadrius collaris*)]. We have obtained samples from several other migratory shorebird species; however, among these others, none of the species that are most abundant in wet open‐field habitats around the Lagoa do Peixe National Park [eg, the buff‐breasted sandpiper (*Calidris subruficollis*)*,* lesser yellowlegs (*Tringa flavipes*), and American golden plover (*Pluvialis dominica*)] have yielded detectable virus. Because these species prefer more open fields, they do not interact much with the other aquatic shorebird species, such as *C. canutus* and *C. fuscicollis*, in which AIVs have been detected. In the case of *S. hirundo*, we found AIV in one of the 2 individuals sampled, because this species is associated with the bodies of water where other aquatic species are found. The fact that all of our positive samples were collected in the austral autumn suggests that the infection builds up during the wintering period, leading to a local outbreak phenomenon, as has been suggested to occur at Delaware Bay.^15^

In 2014, researchers reported the result of a study of the blue‐winged teal and AIV infections in North and South America. They found no evidence of South American lineage genes in samples from these birds, although this teal species is widely distributed in South America, and they suggested that viral gene flow may be limited in some species as a result of geographic barriers.^21^ The major caveat to this interpretation is that we have enough genetic information for comparison purposes from only the H6N1 viruses, and it is possible that both the North and South American lineages circulate at Lagoa do Peixe National Park. Consequently, we are attempting to gather more genetic information from other samples through enrichment procedures. The H11N9 virus is of particular interest, as this subtype has been found in ruddy turnstones (*Arenaria interpres*) in the Amazon region of Brazil, and it has a strong relation to North American AIV lineages, clearly showing that virus exchange between the 2 continents does occur.^20^

In South America, AIV has been confirmed in wild birds in multiple years in Argentina (H13N9, H1N1, H6N2, and H9N2),^13^, ^39^, ^40^ Bolivia (H7N3),^41^ (H7N3),^42^ Colombia (H5N2),^19^ Peru (H3N8, H4N5, H10N9, and H13N2),^42^, ^43^ and Brazil (H11N9).^20^ However, the H6N1 subtype has not previously been identified in shorebirds. Despite the predominance of H6N1 virus, other important subtypes, such as H2N2, H9N2, and H12N5, were identified in birds in the same region during the same collection period. This was the first time that the H2N2 and H12N5 subtypes were reported in South America.

Most recent outbreaks of HPAI in North American poultry clearly resulted from the introduction of low‐pathogenic precursors into poultry populations and their subsequent evolution to a highly pathogenic form in large poultry farms.^44^ More recently, however, a HPAI virus spread from Asia, most likely through wild bird movement, into poultry in North America^45^. Despite Brazil being considered free of HPAI, surveillance of AIV in migratory birds remains incomplete in that country, and AIV data remain scarce. Although H6N1 viruses are considered low‐pathogenic, an isolate of this subtype, albeit genetically distinct from our isolates, has been isolated from a human infection in Taiwan.^46^ Regardless of the zoonotic potential of the viruses found in Lagoa do Peixe National Park, increased surveillance in all animal species in the country is prudent.

The aim of the present work was to assess the importance of Lagoa do Peixe National Park as a source of AIV and to determine the nature of any viruses identified. We were able to detect AIV in the avian community over multiple years, with multiple subtypes being identified. Our phylogenetic analysis demonstrated the high genomewide similarity of our H6N1 isolates and their close relation to South American H6 viruses. Further studies are needed to explain the data showing the presence of viruses of South American lineage in birds that were beginning to migrate north. The avian surveillance program in Brazil is not continuous, but it should be. Our findings are potentially important for surveillance of AIV in Brazil, and they represent a valuable contribution to the study of AIV viruses in South America.

## FIELD TEAM MEMBERS

We thank the Field team: Ana L. Bezerra, Aparecida B. Basler, César R. Santos, Elisa S. Petersen, Emily T. Moura, Fernanda L. Valls, Gabriel V. Vier, Guilherme P. Cauduro, Hélio A. A. Fracasso, Janete F. M. Scherer, Jaqueline Brummelhaus, Jéssica Sartor, Julia V. G. Finger, Laura A. Lindenmeyer de Sousa, Liana C. Rossi, Lucas Kruger, Marina M. M. Seixas, Carla Meneguin, Alexandre Farias de Lemos, Priscilla S. Kiscporski, Roberta C. Piuco, and Suzana Seibert.

## CONFLICT OF INTEREST

None of the authors have a conflict of interest.

## BIOGRAPHICAL SKETCH

Dr. Jansen Araujo has a Ph.D. in Microbiology and is the coordinator of the field team in the Virology Laboratory at the Biomedical Science Institute at the University of São Paulo. His research interests include eco‐epidemiological studies of wildlife and emerging infectious diseases with zoonotic potential in Brazil.

## STATEMENT

Wild birds are the natural reservoir for avian influenza viruses (AIVs). Brazil provides stopover and wintering sites for migratory birds. We wished to investigate the potential for influenza A viruses to be transported by birds that migrate annually along with resident species and to determine the subtypes involved and their potential for reassortment. In Brazil, data about AIV subtypes are scarce, and few groups have worked with AIV in wild birds. At present, only AIV H11N9 has been isolated from the Amazon region (in 2014). This study was conducted to elucidate which AIV subtypes were present at an important stopover and wintering site of wild birds in Brazil and the genetic relatedness between viruses in the Northern and Southern Hemispheres. In addition to performing isolation and next‐generation sequencing (NGS), we used a ferret model to generate influenza A antiserum to compare antigenic differences in influenza viruses from the Northern and Southern Hemispheres.
