# Corrigendum

**DOI:** 10.1111/irv.12573

**Published:** 2018-06-17

In Volume 12, issue 2, 2018, in the article “Migratory birds in Southern Brazil are a source of multiple avian influenza virus subtypes” by Jansen Araujo, Maria Virgínia Petry, Thomas Fabrizio, David Walker, Tatiana Ometto, Luciano M. Thomazelli, Angelo L. Scherer, Patricia P. Serafini, Isaac S. Neto, Scott Krauss, Robert G. Webster, Richard J. Webby, Edison L. Durigon

Below reference had been used incorrectly in the article in the reference section:

46. Wei F, Li J, Ling J, et al. Establishment of SYBR green‐based qPCR assay for rapid evaluation and quantification for anti‐Hantaan virus compounds in vitro and in suckling mice. *Virus Genes*. 2013;46:54‐62.

The correct reference should be

46. Wei S‐H, Yang J‐R, Wu H‐S, et al. Human infection with avian influenza A H6N1 virus: an epidemiological analysis. *Lancet Respir Med*. 2013;1: 771‐778.

The authors apologize for the error.
