# Supplementary material for: Migratory birds in southern Brazil are a source of multiple avian influenza virus subtypes
Source: Influenza Other Respir Viruses. 2017 Dec 15;12(2):220–31. doi: 10.1111/irv.12519 (PMC5820415; doi:10.1111/irv.12519)

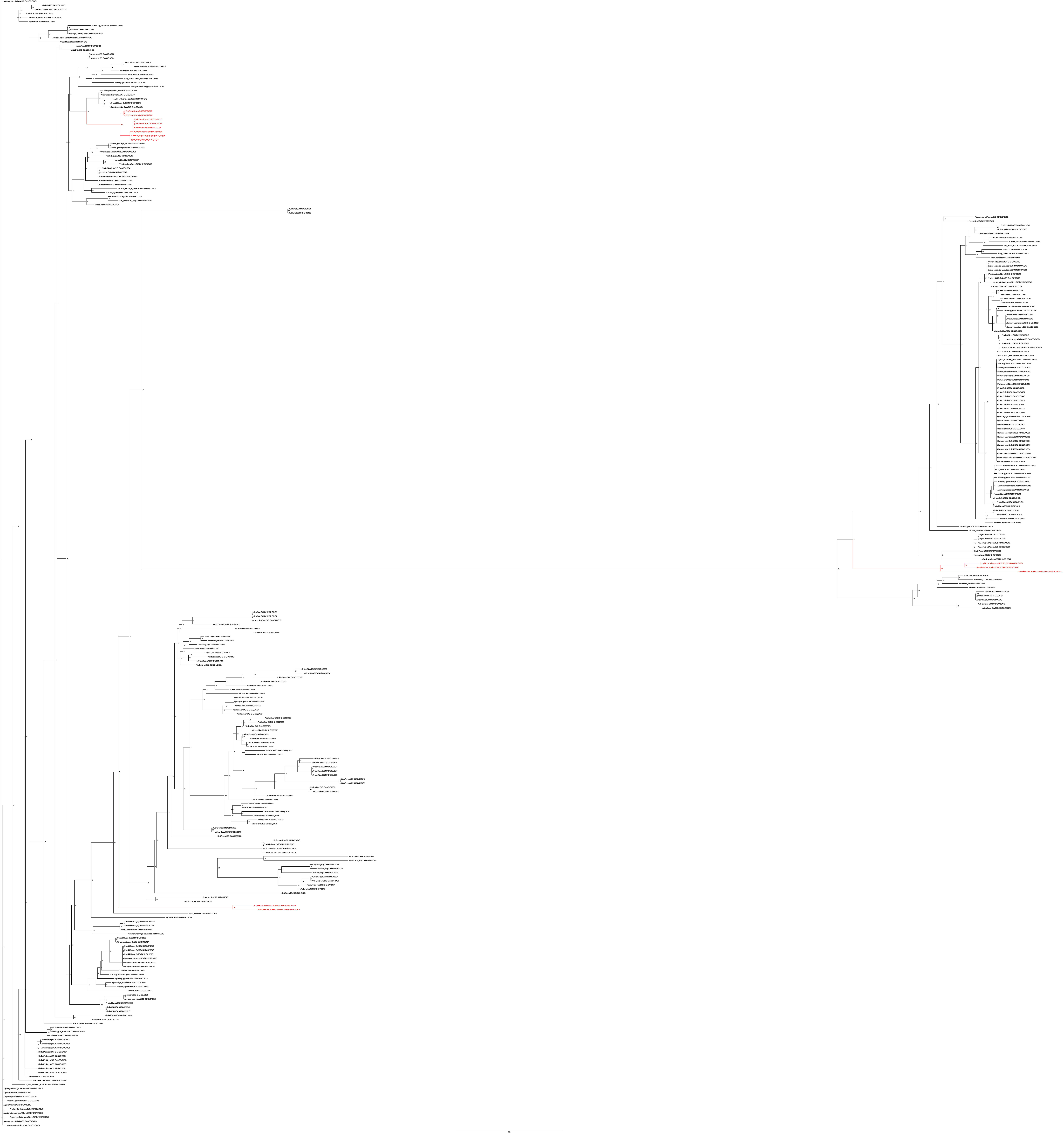

Supplement: Supplementary file 4 [file IRV-12-220-s004.pdf]
